# British Association for the Advancement of Science

**Published:** 1834-04-01

**Authors:** James Yates

**Affiliations:** Secretary to the Council. London


### BRITISH ASSOCIATION FOR THE ADVANCEMENT OF SCIENCE.

*To the Editor of the Medical Quarterly Review.*

Sir: You will oblige me by informing your readers, that the next meeting of the British Association for the Advancement of Science will be held at Edinburgh in the week commencing Monday, September 8th, 1834.

JAMES YATES,
*Secretary to the Council.*

*London, March* 13*th.*

